# Analysis of m6A RNA Methylation-Related Genes in Liver Hepatocellular Carcinoma and Their Correlation with Survival

**DOI:** 10.3390/ijms22031474

**Published:** 2021-02-02

**Authors:** Yong Li, Dandan Qi, Baoli Zhu, Xin Ye

**Affiliations:** 1 Key Laboratory of Pathogenic Microbiology and Immunology, Institute of Microbiology, Chinese Academy of Sciences, Beijing 100101, China; liyrichard@im.ac.cn (Y.L.); zhubaoli@im.ac.cn (B.Z.); 2 Savaid Medical School, University of Chinese Academy of Sciences, Beijing 100049, China; qidd@im.ac.cn

**Keywords:** m6A modification, m6A related genes, survival, liver cancer, TCGA

## Abstract

N6-methyladenosine (m6A) modification on RNA plays an important role in tumorigenesis and metastasis, which could change gene expression and even function at multiple levels such as RNA splicing, stability, translocation, and translation. In this study, we aim to conduct a comprehensive analysis on m6A RNA methylation-related genes, including m6A RNA methylation regulators and m6A RNA methylation-modified genes, in liver hepatocellular carcinoma, and their relationship with survival and clinical features. Data, which consist of the expression of widely reported m6A RNA methylation-related genes in liver hepatocellular carcinoma from The Cancer Genome Atlas (TCGA), were analyzed by one-way ANOVA, Univariate Cox regression, a protein–protein interaction network, gene enrichment analysis, feature screening, a risk prognostic model, correlation analysis, and consensus clustering analysis. In total, 405 of the m6A RNA methylation-related genes were found based on one-way ANOVA. Among them, DNA topoisomerase 2-alpha (*TOP2A*), exodeoxyribonuclease 1 (*EXO1*), ser-ine/threonine-protein kinase Nek2 (*NEK2*), baculoviral IAP repeat-containing protein 5 (*BIRC5*), hyaluronan mediated motility receptor (*HMMR*), structural maintenance of chromosomes protein 4 (SMC4), bloom syndrome protein (*BLM*), ca-sein kinase I isoform epsilon (*CSNK1E*), cytoskeleton-associated protein 5 (*CKAP5*), and inner centromere protein (*INCENP*), which were m6A RNA methylation-modified genes, were recognized as the hub genes based on the protein–protein interaction analysis. The risk prognostic model showed that gender, AJCC stage, grade, T, and N were significantly different between the subgroup with the high and low risk groups. The AUC, the evaluation parameter of the prediction model which was built by RandomForest, was 0.7. Furthermore, two subgroups were divided by consensus clustering analysis, in which stage, grade, and T differed. We identified the important genes expressed significantly among two clusters, including uridine-cytidine kinase 2 (*UCK2*), filensin (*BFSP1*), tubulin-specific chaperone D (*TBCD*), histone-lysine N-methyltransferase PRDM16 (*PRDM16*), phosphorylase b ki-nase regulatory subunit alpha (*PHKA2*), serine/threonine-protein kinase BRSK2 (*BRSK2*), Arf-GAP with coiled-coil (*ACAP3*), general transcription factor 3C polypep-tide 2 (*GTF3C2*), and guanine nucleotide exchange factor MSS4 (*RABIF*). In our study, the m6A RNA methylation-related genes in liver hepatocellular carcinoma were analyzed systematically, including the expression, interaction, function, and prognostic values, which provided an important theoretical basis for m6A RNA methylation in liver cancer. The nine important m6A-related genes could be prognostic markers in the survival time of patients.

## 1. Introduction

Liver hepatocellular carcinoma (LIHC) is one of the common malignant tumors of the digestive system, mainly caused by hepatitis virus infection. It is a leading cause of cancer-related deaths worldwide, with few effective therapeutic options [1]. Liver hepatocellular carcinoma is insidious and develops rapidly, and most patients have already progressed to the middle and late stages when they are diagnosed, and have lost the opportunity for surgery [2]. Therefore, finding new treatment and prognostic targets for LIHC will be beneficial to patients.

Previous studies have shown that tumors are mainly driven by gene mutations [3]. With the deepening of research, epigenetic modifications such as DNA methylation, histone acetylation, and RNA modification have all been proved to participate in the development and progression of tumors, which have been recognized as new treatment and prognostic targets. More and more post-transcriptional modifications of RNA have been identified until now [3]. m6A is considered to be the most common and conservative modification of eukaryotic mRNA, miRNA, and lncRNA. The dynamic balance of m6A methylation modification is maintained in the cell by m6A RNA methylation writers, readers, and erasers [4]. Methyltransferase-like 3 (METTL3), Methyltransferase-like protein 14 (METTL14), WT1-associated protein (WTAP), and RNA binding motif protein 15 (RBM15) were considered as the important m6A RNA methylation writers [5]. Fat mass and obesity-associated protein (FTO) and RNA demethylase ALKBH5 (ALKBH5) were found as demethylases for m6A-modified RNA [6]. The YTH protein family, ETS-related transcription factor Elf-3 (ELF3), Insulin-like growth factor 2 mRNA-binding protein (IGF2BP), and Heterogeneous nuclear ribonucleoprotein (HNRNP) could decode methylation and generate functional signals [7]. In previous studies, m6A modification of RNA were proved to play an important role in tumorigenesis and metastasis. Wen et al. found that m6A on ncRNA NEAT1-1 plays a critical role in the pathogenesis and development of bone metastatic prostate cancer [8]. Chang et al. demonstrated that overexpression of YTH domain-containing family protein 3 (YTHDF3) could induce the transcription of m6A-enriched genes to promote breast cancer metastasis [9]. In addition, Geng et al. systematically analyzed the relation between m6A RNA methylation-related genes and the survival of pancreatic cancer [10].

In our study, we aimed to analyze the data from TCGA to identify the important m6A RNA methylation-related genes in LIHC and evaluate the interaction, function, and prognostic value of the important m6A RNA methylation-related genes in liver hepatocellular carcinoma. Meanwhile, we tried to build a prognostic model to predict the survival status of patients with LIHC.

## 2. Results

### 2.1. Identification of m6A-Related Genes which were Correlated with AJCC Stage

**Figure 1:**
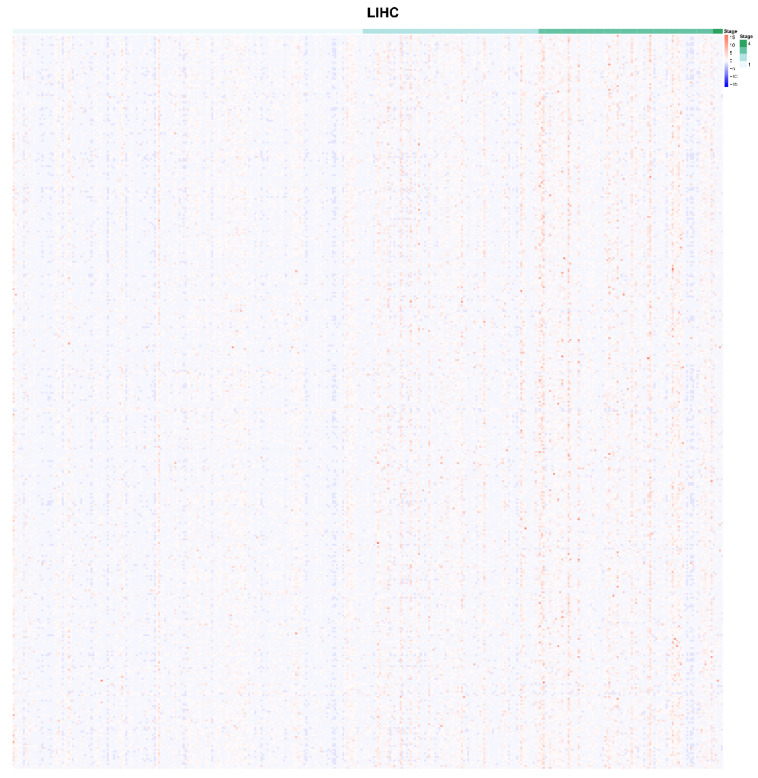
Expression of 405 of m6A RNA methylation-related genes at different stages (I–IV) of liver hepatocellular carcinoma from TCGA dataset.

All 1190 m6A RNA methylation-related genes, which were related to LIHC, were specifically selected with AJCC stage and RNA-seq expression data. A total of 405 m6A RNA methylation-related genes were obtained according to the subgroups which were clustered by AJCC stage. These m6A RNA methylation-related genes were expressed differently at different clinical stages of liver cancer. The different expression of candidate m6A RNA methylation-related genes at different AJCC stages are shown in Figure 1. Obviously, there are nine m6A RNA methylation regulatory factors that differed significantly between the subgroups: *METTL3*, Methyltransferase-like 16 (*METTL16*), *WTAP*, *RBM15*, YTH domain containing 1 (*YTHDC1*), YTH N6-methyladenosine RNA binding protein 1 (*YTHDF1*), YTH N6-methyladenosine RNA binding protein 2 (*YTHDF2*), Heterogeneous nuclear ribonucleoprotein C (*HNRNPC*), and *FTO*, including “writer”, “reader”, and “eraser”.

### 2.2. Functional Annotation of the Survival-Related m6A RNA Methylation-Related Genes

**Figure 2:**
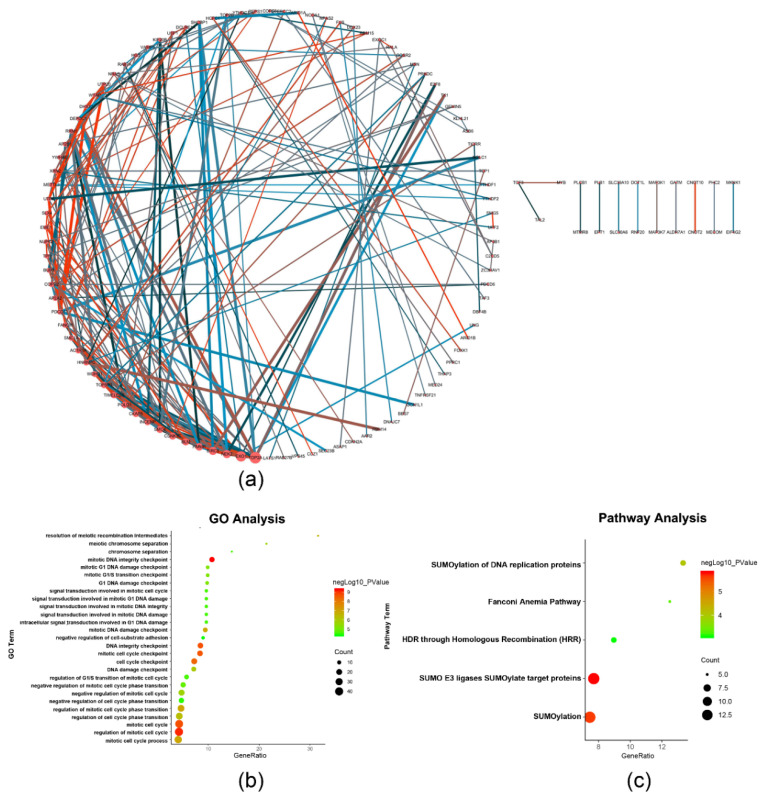
Protein–protein interaction network and enriched analysis of the survival-related m6A RNA methylation-related genes. (**a**) The protein–protein interaction network of the survival-related m6A RNA methylation-related genes was constructed by Cytoscape. Nodes were represented as genes and the size of nodes represents degree of the nodes. Edges were represented as interactions and the color and width of edges represent the value of combined score and co-expression. (**b**) Each biological function term is represented by a node, its color represents *p*-value, and its size represents the number of genes. (**c**) Each pathway term is represented by a node, its color represents *p*-value, and its size represents the number of genes.

The univariate analysis was performed to analyze the relationship between m6A RNA methylation-related genes and survival time of the liver cancer patients from TCGA. A total of 254 of the survival-related genes were obtained from the candidate m6A RNA methylation-related genes, which were significantly related to survival time (*p* < 0.05), including m6A RNA methylation regulatory factors *METTL3*, *WTAP*, *RBM15*, *YTHDC1*, *YTHDF1*, *YTHDF2*, and *HNRNPC*. The above genes are detrimental to the overall survival rate of patients (HR > 1), which are shown in Supplementary Figure S1. The protein–protein interaction network of the survival-related genes was constructed by STRING to identify the hub genes in the PPI network, which are shown in Figure 2a. The hub genes occupied the key nodes in the PPI network and played important roles in the PPI network. DNA topoisomerase 2-alpha (*TOP2A*), exodeoxyribonuclease 1 (*EXO1*), serine/threonine-protein kinase Nek2 (*NEK2*), baculoviral IAP repeat-containing protein 5 (*BIRC5*), hyaluronan mediated motility receptor (*HMMR*), structural maintenance of chromosomes protein 4 (*SMC4*), bloom syndrome protein (*BLM*), casein kinase I isoform epsilon (*CSNK1E*), cytoskeleton-associated protein 5 (*CKAP5*), and inner centromere protein (*INCENP*) were identified as the hub genes based on the degrees of nodes which were more than 10 (Figure 2a) [11]. *TOP2A*, *EXO1*, *NEK2*, *BIRC5*, *SMC4*, *BLM*, *CSNK1E*, *CKAP5*, and *INCENP* were related to DNA replication and cell division, and HMMR was related to cell motility [12,13,14,15,16,17,18,19,20,21,22,23,24]. The detailed functions of the hub genes are described in the discussion section and Supplementary Table S3. Enrichment analysis was performed by ClueGO, including Gene Ontology (GO) and Pathway, which are shown in Figure 2b,c. The results show that the biological functions, such as mitotic DNA integrity checkpoint, regulation of mitotic cell cycle, and mitotic DNA damage checkpoint, were related to the cell proliferation and cell cycle. In addition, SUMOylation of DNA replication proteins, HDR through Homologous Recombination (HRR), the Fanconi Anemia Pathway, SUMOylation, and SUMO E3 ligases SUMOylate target proteins were indicated the most significantly enriched (all *p* < 0.001).

### 2.3. The Prognostic Value of the Survival-Related m6A RNA Methylation-Related Genes and a Risk Signature Built Using Nine Selected Survival-Related m6A RNA Methylation-Related Genes

**Figure 3:**
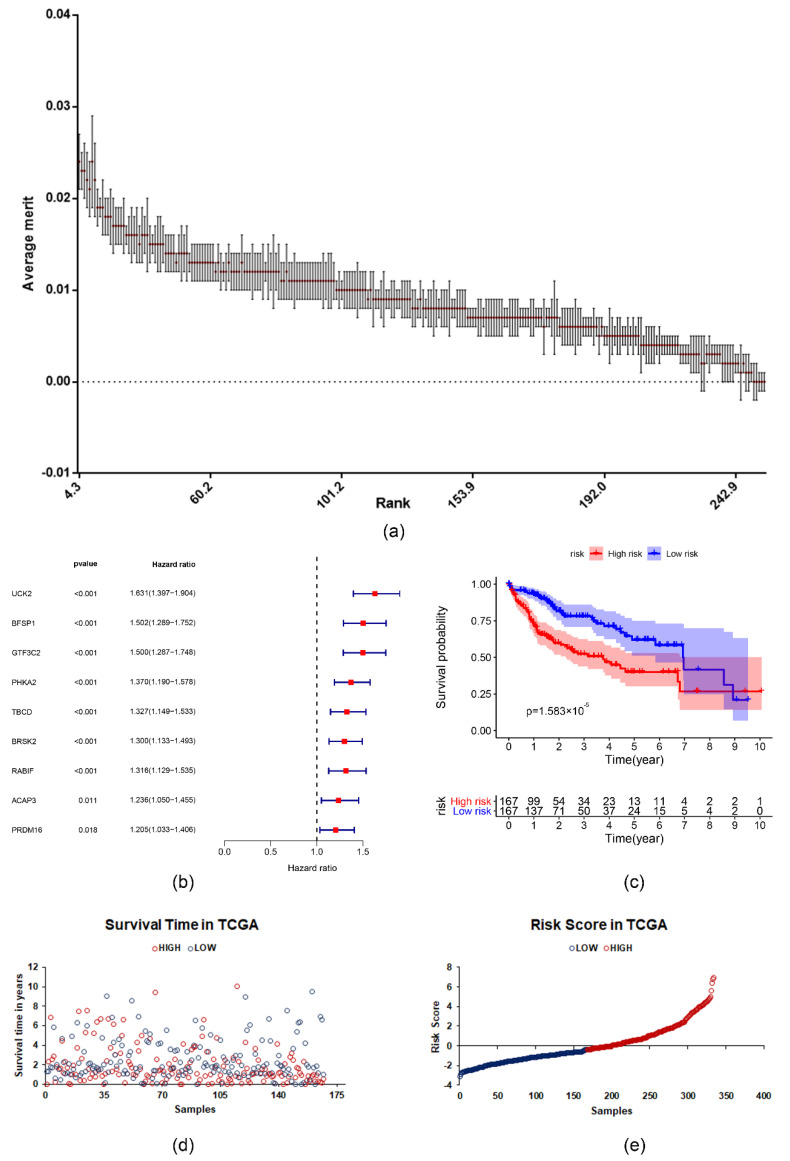
Features selection and Cox regression analysis were used to construct and analyze risk scores based on the survival-related m6A RNA methylation-related genes. (**a**) Rank of the survival-related m6A RNA methylation-related genes based on Relief algorithm. (**b**) The impact of important survival-related m6A RNA methylation-related genes on overall survival. (**c**) The Kaplan–Meier curve described the significant survival difference between the high-risk group and the low-risk group. (**d**) The distribution of survival time of samples in TCGA dataset. (**e**) The distribution of risk score of samples in TCGA dataset.

A relief algorithm was used to filter the important survival-related genes (Figure 3a). Nine of the m6A RNA methylation-related genes from the LIHC cohort from TCGA inversely related to survival were chosen for further analysis. They are uridine-cytidine kinase 2 (*UCK2*), filensin (*BFSP1*), tubulin-specific chaperone D (*TBCD*), histone-lysine N-methyltransferase PRDM16 (*PRDM16*), phosphorylase b kinase regulatory subunit alpha (*PHKA2*), serine/threonine-protein kinase BRSK2 (*BRSK2*), Arf-GAP with coiled-coil (*ACAP3*), general transcription factor 3C polypeptide 2 (*GTF3C2*), and guanine nucleotide exchange factor MSS4 (*RABIF*) (Figure 3b). The LIHC cohort was split as high and low risk groups according to the median value of risk score. Kaplan–Meier survival analysis was performed to analyze the differences between the two groups. The result showed the overall survival was significantly better in low-risk patients than high-risk patients (*p* = 1.583 × 10^−5^) (Figure 3c–e). Then, the data of GSE76427 were used to test the performance of risk score. The results are shown in Supplementary Figure S2.

**Figure 4:**
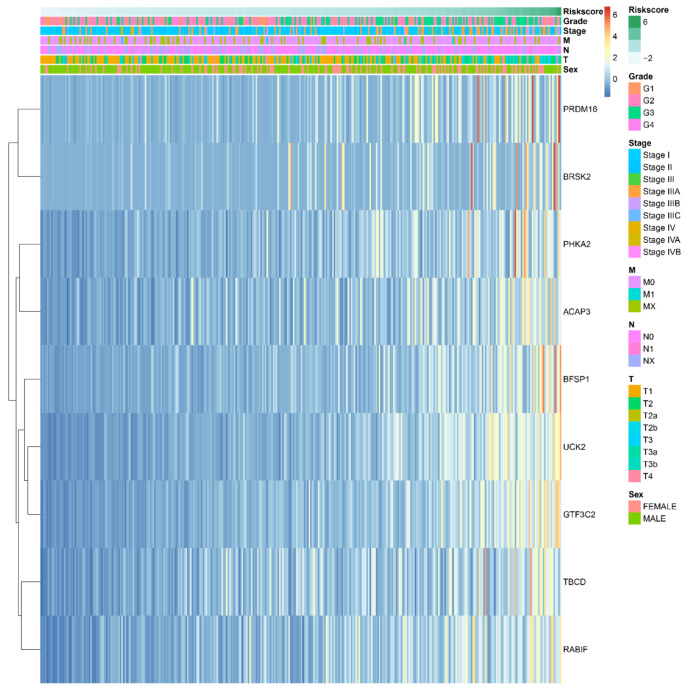
The heatmap of the expression of 9 survival-related m6A RNA methylation-related genes in the different risk groups. The distribution of clinical features was compared in the different risk group.

**Figure 5:**
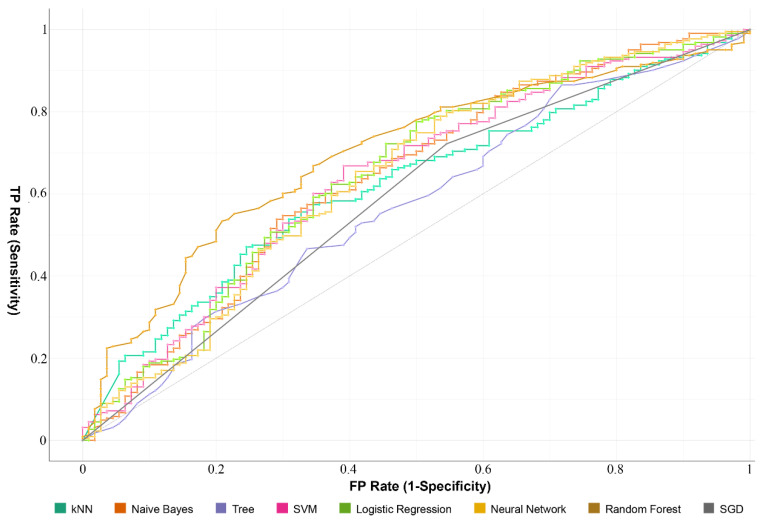
ROC curves showed the predictive efficiency of the prognostic model of TCGA dataset.

To explore the correlations between the prognostic model and various clinical features, the clinical features were analyzed with the risk scores. Gender, AJCC stage, grade, T, and N were significantly different between the subgroup with high and low risk scores. Age and M were not significantly different between the high- and low-risk groups. The results are shown in Supplementary Figures S3 and S4. The expression of important survival-related genes and various clinical features of corresponding patients are shown in Figure 4. Based on the important survival-related genes, prediction models were built by different machine learning algorithms, which are shown in Figure 5 and Table 1. The model which was constructed based on RandomForest showed better prediction performance (AUC = 0.70), suggesting it could be used to predict the survival event of LIHC patients, and be used as an indicator to evaluate the prognosis of patients.

**Figure 6:**
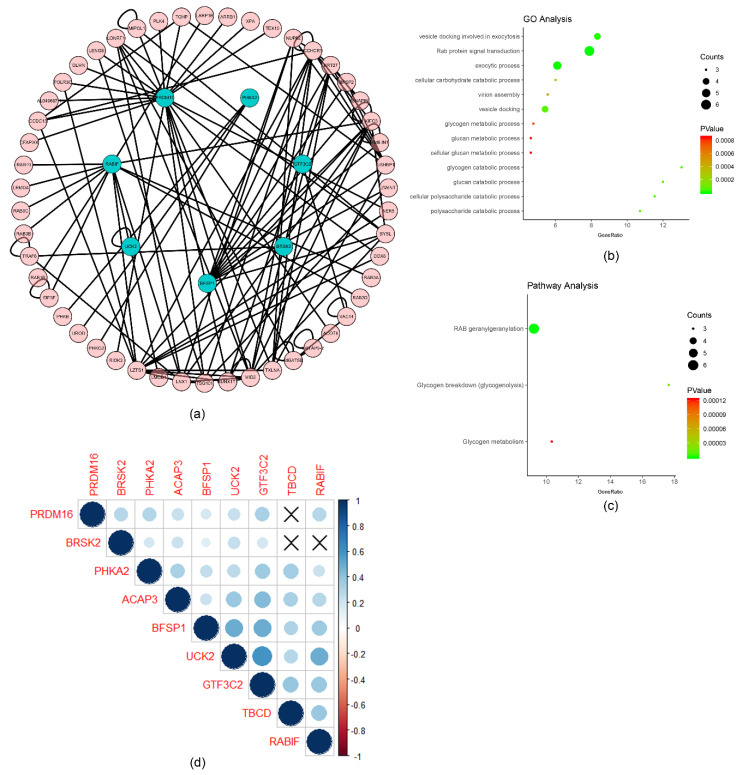
Interactions, functions, and correlations of 9 survival-related m6A RNA methylation-related genes. (**a**) The protein–protein interaction network of 9 m6A RNA methylation-related genes with interacting proteins. (**b**) GO analysis of proteins in (**a**). (**c**) Pathway analysis of proteins in (**a**). (**d**) Spearman correlation analysis of 9 survival-related m6A RNA methylation-related genes.

To better analyze the interactions and functions among the nine important survival-related genes, analysis of interactions and correlations was carried out between these genes. The protein–protein interaction network was constructed based on HuRI, and seven important survival-related genes were obtained in the network (Figure 6a). These genes were related to virion assembly, vesicle docking, exocytic process, vesicle docking involved in exocytosis, Rab protein signal transduction, and cellular carbohydrate catabolic process according to the GO analysis (Figure 6b) and Pathway analysis (Figure 6c). The correlations of the survival-related genes were calculated by Spearman’s correlation significantly, which are shown in Figure 6d. Evidently, *GTF3C2*, *BFSP1*, and *RABIF* were positively associated with *UCK2*, and *BFSP1* was positively associated with *GTF3C2*.

### 2.4. The Prognostic Value of the Important m6A-Related Genes for Liver Hepatocellular Carcinoma with Distinct Clinical Outcomes

**Figure 7:**
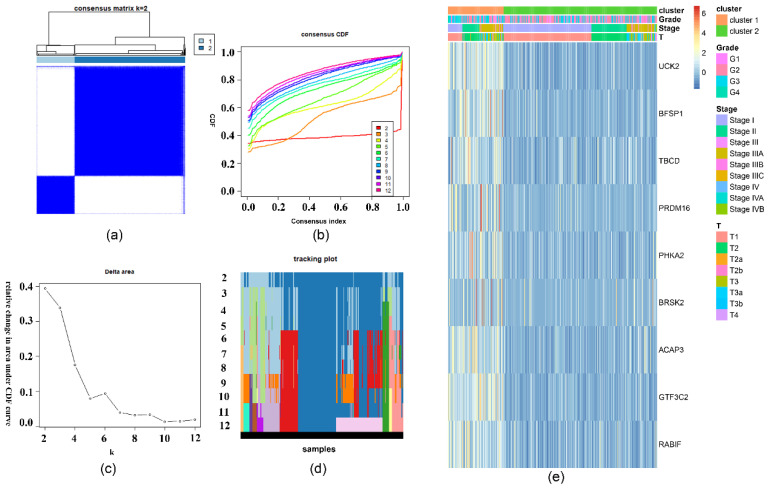
TCGA samples were divided into 2 subtypes by using 9 survival-related m6A RNA methylation-related genes. (**a**) The consensus clustering matrix at *k* = 2. (**b**) CDF curve for k = 2 to 12. (**c**) The relative variation of the area under the CDF curve that k from 2 to 12. (**d**) Tracking plot for k from 2 to 12. (**e**) The heatmap of the expression of 9 survival-related m6A RNA methylation-related genes in the different risk groups. The distribution of clinical features was compared in the different risk group.

**Figure 8:**
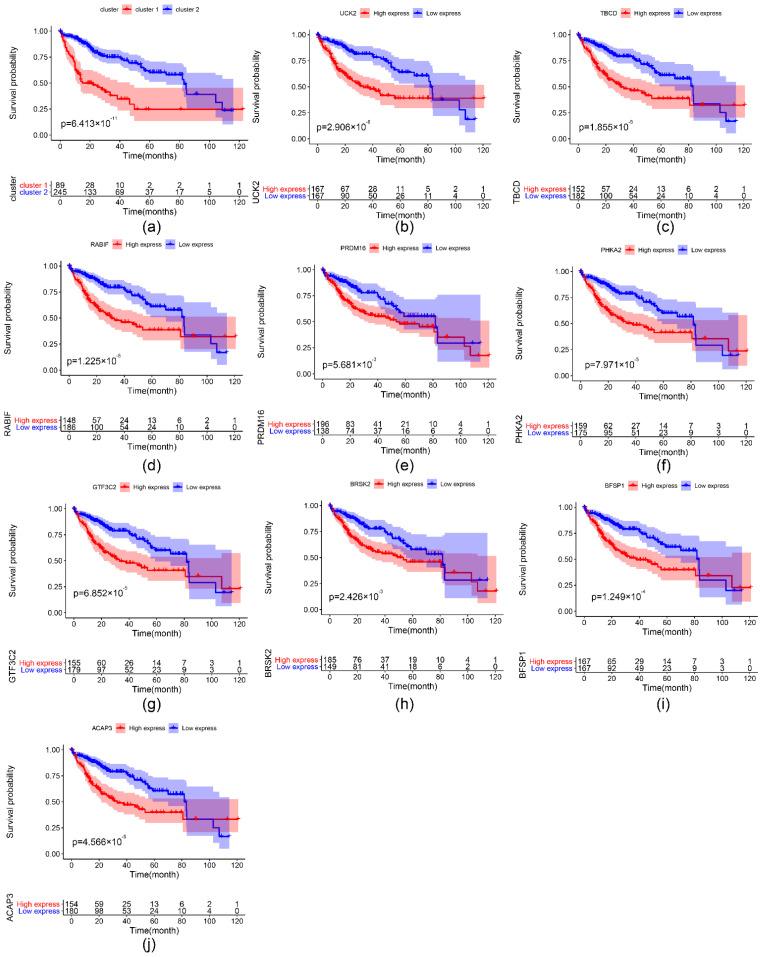
Kaplan–Meier analysis of clusters according to the ConsensusClusterPlus and the important survival-related m6A RNA methylation-related genes. (**a**) Kaplan–Meier curves of TCGA samples which were divided into 2 clusters according to the ConsensusClusterPlus. (**b**–**j**) Kaplan–Meier curves of the important survival-related m6A RNA methylation-related genes. The unit of survival time is months.

To specifically analyze the prognostic value of the nine important m6A-related genes in liver hepatocellular carcinoma, TCGA liver hepatocellular carcinoma samples were divided into different subgroups according to the expression of the nine selected survival-related m6A RNA methylation-related genes by using the R package ConsensusClusterPlus. K = 2 should be an adequate choice according to the expression similarity of the important m6A-related genes with clustering stability from 2 to 12 based on the TCGA (Figure 7a–d). The clinical features differed significantly among cluster 1 and cluster 2 when they were analyzed based on the Kolmogorov–Smirnov test, stage, grade, and T (Figure 7e). In addition, there is a clear difference in survival time between the two clusters (*p* = 6.413 × 10^−11^) (Figure 8a). The expression of the nine selected survival-related m6A RNA methylation-related genes was significantly different (*p* < 0.05, Supplementary Figure S4). These genes were associated with poor survival, which are shown in Figure 8b–j.

## 3. Discussion

Liver hepatocellular carcinoma is the malignant tumor with high mortality rate in the world. At present, the mechanism of LIHC development is still not fully understood [25]. The prognosis of patients is poor. Surgical interventions and other treatment methods often bring greater damage to patients and may not be effective to extend patient survival time. Therefore, looking for treatment and prognosis targets of LIHC can benefit more LIHC patients and it is easier to realize current personalized treatment and minimally invasive or even non-invasive treatment [26]. Previous studies pointed out that tumors including LIHC were driven by genetic mutations and were a kind of genetic disease. However, DNA methylation, miRNA, lncRNA, histone acetylation, and others have been confirmed to participate in tumorigenesis and development [4]. A total of 163 different chemical modifications of RNA were identified before 2018. m6A is considered to be the most common, and most conservative modification of eukaryotic messenger RNA (mRNAs), miRNA, and lncRNA. Some studies point out that m6A modification is related to the occurrence and development of liver hepatocellular carcinoma. METTL3 is significantly upregulated in liver cancer. Overexpression of METTL3 could promote HCC proliferation and migration. METTL3 could regulate the m6A modification of SOCS2 mRNA and contributes to LIHC process. METTL3 is also a tumor suppressor—it promotes the occurrence and development of liver cancer by reducing the stability of SOCS2 mRNA through m6A-YTHDF2-dependent pathways [27]. In addition, YTHDF2 is closely related to the degree of malignancy of liver cancer, and regulates mRNA degradation by recognizing m6A sites, resulting in the enhancement of liver cancer cell proliferation [28]. Those studies prove that m6A modification could have an important impact on the occurrence and development of liver hepatocellular carcinoma.

METTL3, METTL14, FTO, and ALKBH5 play important roles in the development and progression of liver hepatocellular carcinoma in previous studies [29,30,31,32]. However, previous studies only reported that they influence the mRNA level of target genes individually, and their role in predicting prognostic and survival time of LIHC patients as a cluster is still unexplored. Therefore, we carried out studies to identify the important survival-related genes from 1190 m6A RNA methylation-related genes and analyzed the relations between genes and the clinical features of LIHC patients.

A total of 405 m6A RNA methylation-related genes were obtained according to the analysis of different genes across different AJCC stages, which should be related to the development and progression of LIHC. It is worth nothing that METTL3, METTL16, WTAP, RBM15, YTHDC1, YTHDF1, YTHDF2, HNRNPC, and FTO, which were the m6A RNA methylation regulatory factors, were identified to have significant differences among AJCC-stage subgroups.

METTL3 with catalytic m6A formation function has been proved to play an important role in tumors, brain development, and regulation of stem cell fate [33]. Current evidence has shown that METTL3 expression is upregulated and mutes SOCS2 through the m6a-dependent pathway to promote LIHC progression [29]. Other studies showed EIF3 is recruited by METTL3 into the translation initiation complex to enhance the translation of target mRNA, such as epidermal growth factor receptor (EGFR) and Tafazzin (TAZ) [34]. METTL16 was proved to control SAM homeostasis by regulating the abundance of SAM synthase MAT2A mRNA in response to changing intracellular SAM levels [35]. WTAP could specifically bind to the WT1 gene in vivo and in vitro, which could participate in a variety of normal physiological processes in the body such as m6A methylation modification, RNA alternative splicing, cell cycle regulation, and play an important regulatory role in the occurrence and development of various malignant tumors of which we know little [36]. RBM15 could recognize and interact with NXF1 to assist in promoting the expression of genes, which could participate in the output pathway [37]. The YTH protein family has a homologous YTH domain used to recognize the m6a site, which belong to “Readers”. YTHDF1 promoted mRNA stability and translation, and YTHDF2 promotes RNA degradation by selectively binding to target RNA decay sites in current studies. In addition, YTHDC1 could participate in RNA variable cleavage [38]. RNA-HNRNPC could be modified by m6A after transcription to regulate the interaction to affect the structure of coding and non-coding RNA [39]. FTO, which is identified as the first m6aRNA demethylase, could promote LIHC progression by mediating PKM2 demethylation with the high expression of FTO according to recent studies [35]. In addition, METTL3, WTAP, RBM15, YTHDC1, YTHDF1, YTHDF2, and HNRNPC were found to have a significant relation with survival time in our research.

A total of 254 survival-related genes were obtained from the 405 candidate m6A RNA methylation-related genes according to the univariate analysis. The results of protein–protein interaction analysis between 254 survival-related genes point to the fact that *TOP2A*, *EXO1*, *NEK2*, *BIRC5*, *HMMR*, *SMC4*, *BLM*, *CSNK1E*, *CKAP5*, and *INCENP* are identified as the hub genes in the network.

The role of TOP2A is to mediate DNA double strand unwinding, which was recognized as a drug target to design drugs, such as Etoposide. The recent studies showed overexpression of TOP2A is associated with early onset, changes in drug treatment response, and poor prognosis, and indicates that TOP2A gene amplification may be the internal mechanism that causes these changes [12,13,14].

EXO1 genes which are related to mismatch repairing were recognized as the new cancer driver genes in a recent study, which are closely related to m6A RNA methylation-related genes [15].

Furthermore, current reports have showed NEK2, as an important cyclin, has the functions of regulating the assembly and separation of centrosomes, the formation of spindles, and the precise separation of chromosomes [16,17]. In addition, NEK2 may also become an important marker for tumor differentiation and prognosis detection, and is an important potential target for cancer treatment [18].

BIRC5 was located at the center of the signaling networks of tumors, which was regulated by kinases and enzymes, such as AURKB, CDK1, and HSP90. BIRC5 also regulated the expression of proteins to affect the functions of tumors, such as IKK-β, caspase3, and HIF1A. HMMR is significantly overexpressed in patients with primary liver cancer and promotes the proliferation of liver cancer cells, which was reported in recent studies [19,20].

SMC4 and SMC2 form the SMC2–SMC4 dimer to participate in cell mitosis, gene regulation, and DNA repair, and are closely related to tumorigenesis. BLM, as a member of the RecQ-like helicase family, plays an important role in cellular metabolism such as DNA replication, recombination, transcription, repair, and telomere maintenance, which overexpressed in the tumor cell [21]. CSNK1E was identified as a circadian gene, which has been proved to be related to the DNA replication and repair that was mediated by β-catenin in previous studies [22]. CKAP5 belong to the microtubule-like protein family, which is involved in the assembly of microtubules. The overexpression of CKAP5 could promote the development of tumor [23]. INCENP could be used as a structural platform to complete the assembly of the CPC’s core structure and participate in the activation process of Aurora B kinase [24].

To further analyze the prognostic potential of survival-related m6A RNA methylation-related genes, we used the Relief algorithm to filter the important genes and constructed a risk prognostic model. The high-risk and low-risk groups had significant differences in overall survival, and gender, AJCC stage, grade, T, and N had significant differences based on different groups in further analysis. Then, we built a different prognosis model based on different algorithms, and the model which was constructed by RandomForest had the best evaluation values, an AUC more than 0.7, which shows that the model could predict the survival of LIHC patients with high accuracy. We also analyzed the interactions of the important genes based on the HuRI, and we obtained the function of those genes, which were related to the cellular carbohydrate catabolic process and vesicle docking. Correlation analysis showed *GTF3C2*, *BFSP1*, and *RABIF* were positively associated with *UCK2* and *BFSP1* was positively associated with *GTF3C2*. However, we need more works to understand the interaction of the nine important survival-related m6A RNA methylation-related genes.

Two clusters of liver cancer were identified according to the expression of the nine important survival-related m6A RNA methylation-related genes by consensus clustering analysis. Stage, grade, and T differed significantly among cluster 1 and cluster 2. The nine important survival-related m6A RNA methylation-related genes were identified, which all differed in the subgroups in further studies, and those genes were positively associated with positive cancer status [40,41,42,43,44,45,46]. Although there have been some studies on these genes, more studies need to be performed to explain the mechanism of the nine important survival-related m6A RNA methylation-related genes in liver cancer.

## 4. Materials and Methods

### 4.1. Data Preparation

The RNA sequencing data and clinical information of LIHC patients were downloaded from The Cancer Genome Atlas (TCGA, 2020) [47]. GEO data were downloaded from the GEO database (https://www.ncbi.nlm.nih.gov/geo/). m6A RNA methylation-related genes, which were related to LIHC, were collected from the known literature [48,49,50,51] and were download from the m6Avar database (http://m6avar.renlab.org/) [52]. The m6Avar database was a database of functional variants involved in m6A modification. The m6A RNA methylation-related genes expression data from TCGA were obtained and standardized as shown in Supplementary Table S1. The clinical information of all LIHC patients is summarized in Supplementary Table S2.

### 4.2. The Differentially Expressed Genes (DEGs) Screening and Enrichment Analysis

One-way ANOVA was employed to analyze the difference in expression of m6A RNA methylation-related genes between different stages of liver hepatocellular carcinoma based on the TCGA data. Univariate analysis was performed to analyze the correlation between m6A RNA methylation-related genes and survival. The protein–protein interaction network of survival-related DEGs was formed by STRING (https://string-db.org/) to analyze the key proteins in the network. All statistically enriched terms (Gene Ontology (GO) and Pathway terms) were analyzed by ClueGO.

### 4.3. The Filter of Survival-Related m6A RNA Methylation-Related Genes and the Evaluation of Risk Score

To explore the prognostic potential importance of the survival-related m6A RNA methylation-related gene set, a Relief algorithm was used to filter the important survival-related genes based on the Weka platform (Weka 3.8.4). The Relief algorithm belongs to feature weighting algorithms, which assigned different weight to features according to the correlation of features and categories and features with a weight less than a certain threshold will be removed.

Kaplan–Meier was used to test the significance of the survival curves and analyze the difference in survival between different subgroups. To construct the risk characteristics and calculate the risk score, the formula was as follows:

$$Risk\;Score=\;\overset{n}{\underset{i=1}{\sum }}Coef_{i}\times Exp_{i}$$

According to the median risk score, all of the samples were divided into low and high subtypes. Survival analysis was performed on the low and high subtypes using the R software “survival” package.

To better analyze the interaction and function among the important survival-related genes, an analysis of the interactions was carried out between these genes by using the HuRI (http://www.interactome-atlas.org/) and the correlations were analyzed by using R software (3.6.3).

### 4.4. Construction and Evaluation of Prognostic Models Based on the Important Survival-Related Genes

The important survival-related genes were used to construct a predicted model to predict the prognostics of patients by different machine learning algorithms based on a 20-fold cross-validation method and all setting parameters were set as default based on orange data mining (Orange version: 3.25.0) [53]. According to the different evaluation parameters to evaluate models, the model which had the highest evaluation parameters was selected as the prognostic model.

### 4.5. Consensus Clustering for the Important Survival-Related m6A RNA Methylation-Related Genes

To specifically investigate the function of the candidate m6A RNA methylation-related genes in liver hepatocellular carcinoma, the unsupervised clustering method—the ConsensusClusterPlus R package—was used to cluster the samples. The clusters were used to analyze the candidate m6A-related genes in liver hepatocellular carcinoma.

## 5. Conclusions

In summary, the expression, interaction, function, and prognostic of m6A RNA methylation-related genes in liver cancer were elucidated in our research. In addition, the relations between m6A RNA methylation-related genes and clinical features were found in our study. A number of genes were recognized as potential drug targets, which provide new treatment strategies for the treatment of liver cancer. Our research provides important evidence for further research on the function of RNA m6A methylation and related genes in liver cancer.

## Figures and Tables

**Table 1:** Evaluation results of models based on different algorithms.

Model	AUC	CA	F1	Precision	Recall
RF	0.70	0.67	0.63	0.64	0.67
LG	0.65	0.70	0.64	0.67	0.70
NB	0.64	0.65	0.65	0.64	0.65
NN	0.64	0.70	0.65	0.67	0.70
SVM	0.64	0.69	0.63	0.66	0.69
KNN	0.62	0.66	0.61	0.61	0.66
SGD	0.59	0.64	0.64	0.64	0.64
DT	0.57	0.68	0.64	0.64	0.67

Note: RF—Random Forest; LG—Logistic Regression; NB—Naïve Bayes; NN—Neural Network; SVM—Support Vector Machine; KNN—k-Nearest Neighbor; SGD—Stochastic Gradient Descent; DT—Decision Tree.

## Data Availability

Publicly available datasets were analyzed in this study. This data can be found here: https://portal.gdc.cancer.gov/; https://www.ncbi.nlm.nih.gov/geo/; http://m6avar.renlab.org/. The predict model presented in this study are openly available in https://github.com/RichardYongLi/m6A-prognostic-model-orange.

## Supplementary Materials

Supplementary materials can be found at https://www.mdpi.com/1422-0067/22/3/1474/s1.

## Abbreviations

ACAP3	Arf-GAP with coiled-coil
AJCC	American Joint Committee on Cancer
AUC	Area Under Curve
AURKB	Aurora kinase B
BFSP1	Filensin
BIRC5	Baculoviral IAP repeat-containing protein 5
BLM	Bloom syndrome protein
BRSK2	Serine/threonine-protein kinase BRSK2
CDK1	Cyclin-dependent kinase 1
CKAP5	Cytoskeleton-associated protein 5
CSNK1E	Casein kinase I isoform epsilon
DEGs	Differentially expressed genes
DT	Decision Tree
EGFR	Epidermal growth factor receptor
EXO1	Exodeoxyribonuclease 1
FTO	Fat mass and obesity-associated protein
GO	Gene Ontology
GTF3C2	General transcription factor 3C polypeptide 2
HCC	Hepatocellular Carcinoma
HMMR	Hyaluronan mediated motility receptor
HNRNPC	Heterogeneous nuclear ribonucleoprotein C
HSP90	Heat shock protein HSP 90
INCENP	Inner centromere protein
KNN	k-Nearest Neighbor
LG	Logistic Regression
LIHC	Liver hepatocellular carcinoma
m6A	N6-methyladenosine
METTL16	Methyltransferase like 16
METTL3	Methyltransferase like 3
NB	Naïve Bayes
NEK2	Serine/threonine-protein kinase Nek2
NN	Neural Network
NXF1	Nuclear RNA export factor 1
PHKA2	Phosphorylase b kinase regulatory subunit alpha
PRDM16	Histone-lysine N-methyltransferase PRDM16
RABIF	Guanine nucleotide exchange factor MSS4
RBM15	RNA binding motif protein 15
RF	Random Forest
SGD	Stochastic Gradient Descent
SMC4	Structural maintenance of chromosomes protein 4
SOCS2	Suppressor of cytokine signaling 2
SVM	Support Vector Machine
TAZ	Tafazzin
TBCD	Tubulin-specific chaperone D
TCGA	The Cancer Genome Atlas
TOP2A	DNA topoisomerase 2-alpha
UCK2	Uridine-cytidine kinase 2
WTAP	WT1 associated protein
YTHDC1	YTH domain containing 1
YTHDF1	YTH N6-methyladenosine RNA binding protein 1
YTHDF2	YTH N6-methyladenosine RNA binding protein 2
YTHDF3	YTH N6-methyladenosine RNA binding protein 3
