# Supplementary material for: Analysis of m6A RNA Methylation-Related Genes in Liver Hepatocellular Carcinoma and Their Correlation with Survival
Source: Int J Mol Sci. 2021 Feb 2;22(3):1474. doi: 10.3390/ijms22031474 (PMC7867233; doi:10.3390/ijms22031474)

Supplementary Figure S1

Forest plot of the survival-related m6A RNA methylation related genes. The univariate analysis was performed to analyze the relationship between m6A RNA methylation related genes and survival time of the liver cancer patients from TCGA.

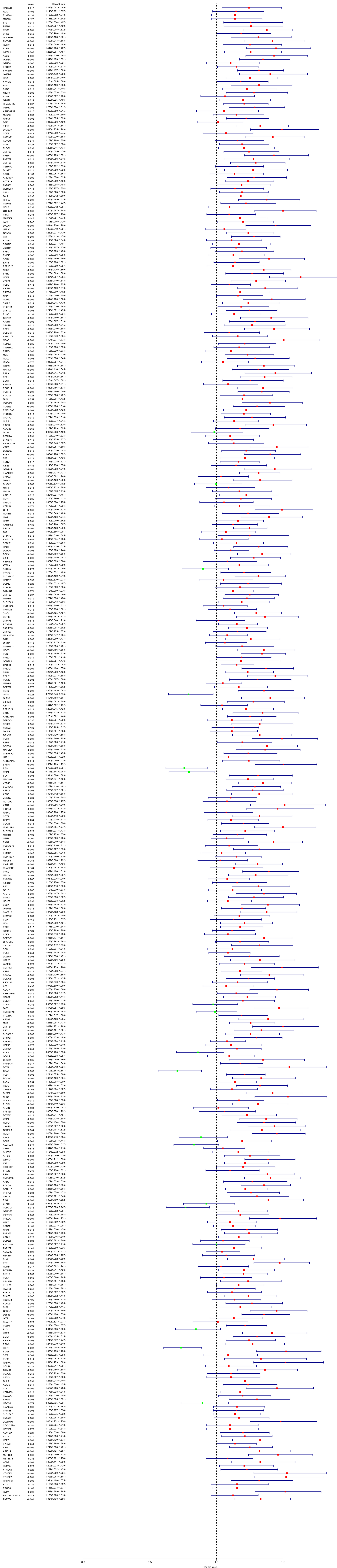

Supplement: Supplementary file 1 [file ijms-22-01474-s001.zip › Supplementary/Supplementary Figure S1.pdf]
