# Supplementary material for: Analysis of m6A RNA Methylation-Related Genes in Liver Hepatocellular Carcinoma and Their Correlation with Survival
Source: Int J Mol Sci. 2021 Feb 2;22(3):1474. doi: 10.3390/ijms22031474 (PMC7867233; doi:10.3390/ijms22031474)

Supplementary Figure S2

The KM curve described the significant survival difference between the high-risk group and the low-risk group of GSE76427.

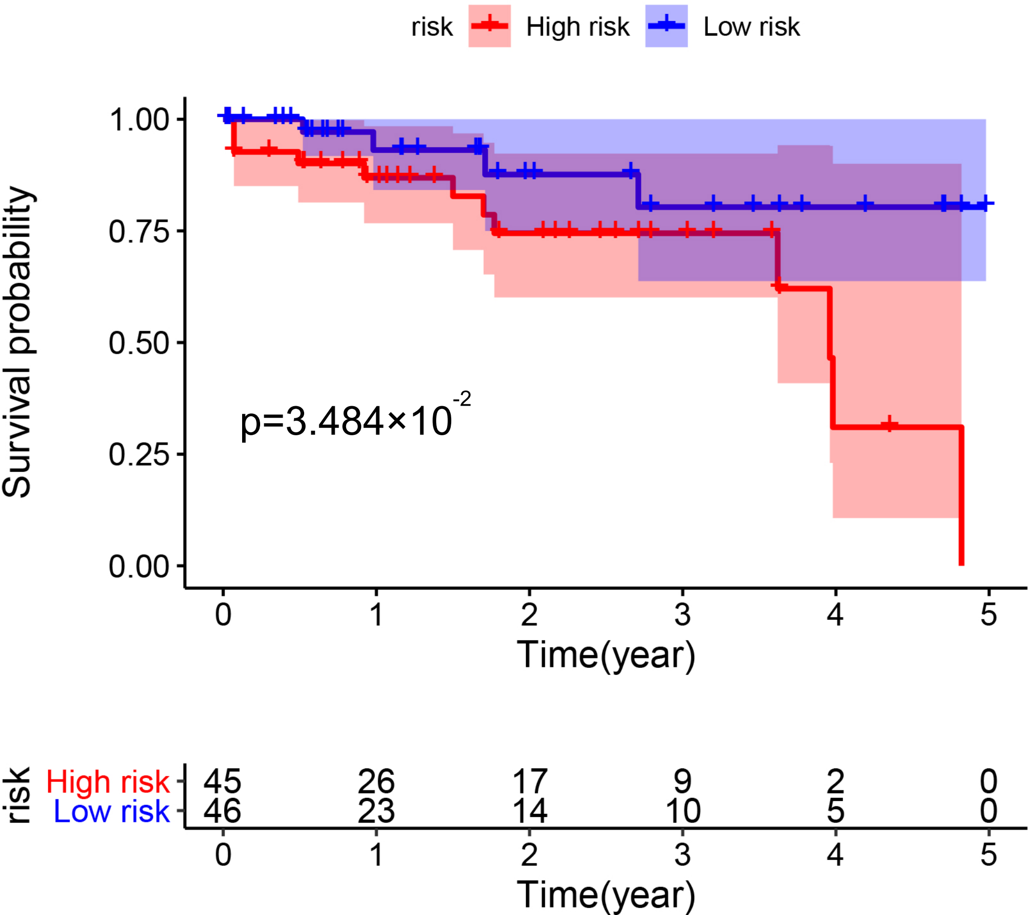

Supplement: Supplementary file 1 [file ijms-22-01474-s001.zip › Supplementary/Supplementary Figure S2-revised.pdf]
