# Supplementary material for: Analysis of m6A RNA Methylation-Related Genes in Liver Hepatocellular Carcinoma and Their Correlation with Survival
Source: Int J Mol Sci. 2021 Feb 2;22(3):1474. doi: 10.3390/ijms22031474 (PMC7867233; doi:10.3390/ijms22031474)

Supplementary Figure S3

The clinical features were analyzed with the risk scores. Gender, AJCC stage, grade, T and N were significantly differed between the subgroup with high and low risk scores. Age and M were not significantly different between the high- and low-risk groups.

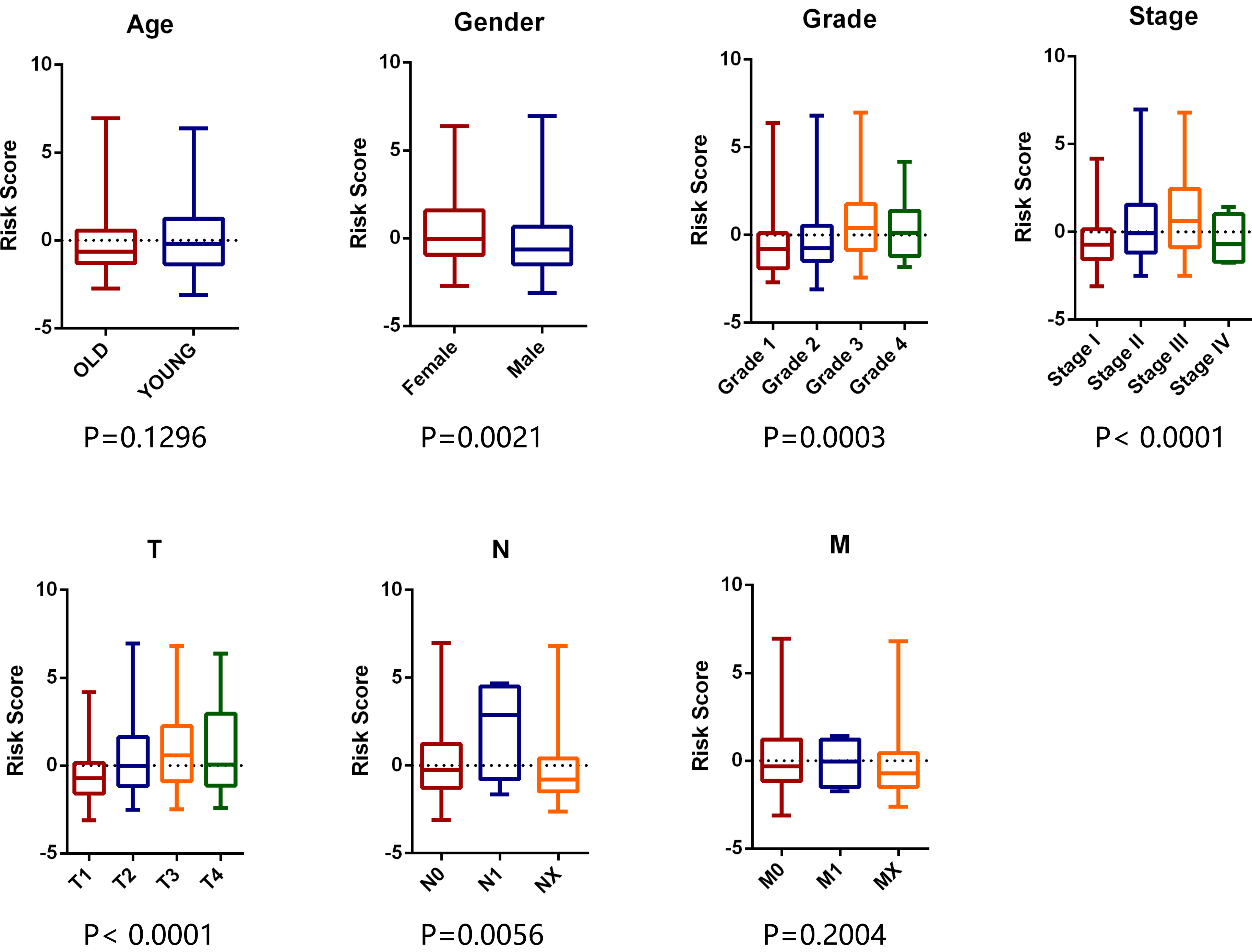

Supplement: Supplementary file 1 [file ijms-22-01474-s001.zip › Supplementary/Supplementary Figure S3.pdf]
