# Supplementary material for: Analysis of m6A RNA Methylation-Related Genes in Liver Hepatocellular Carcinoma and Their Correlation with Survival
Source: Int J Mol Sci. 2021 Feb 2;22(3):1474. doi: 10.3390/ijms22031474 (PMC7867233; doi:10.3390/ijms22031474)

Supplementary Figure S4

The difference expression of genes in clusters. The expression of the nine selected survival-related m6A RNA methylation related genes were significantly different

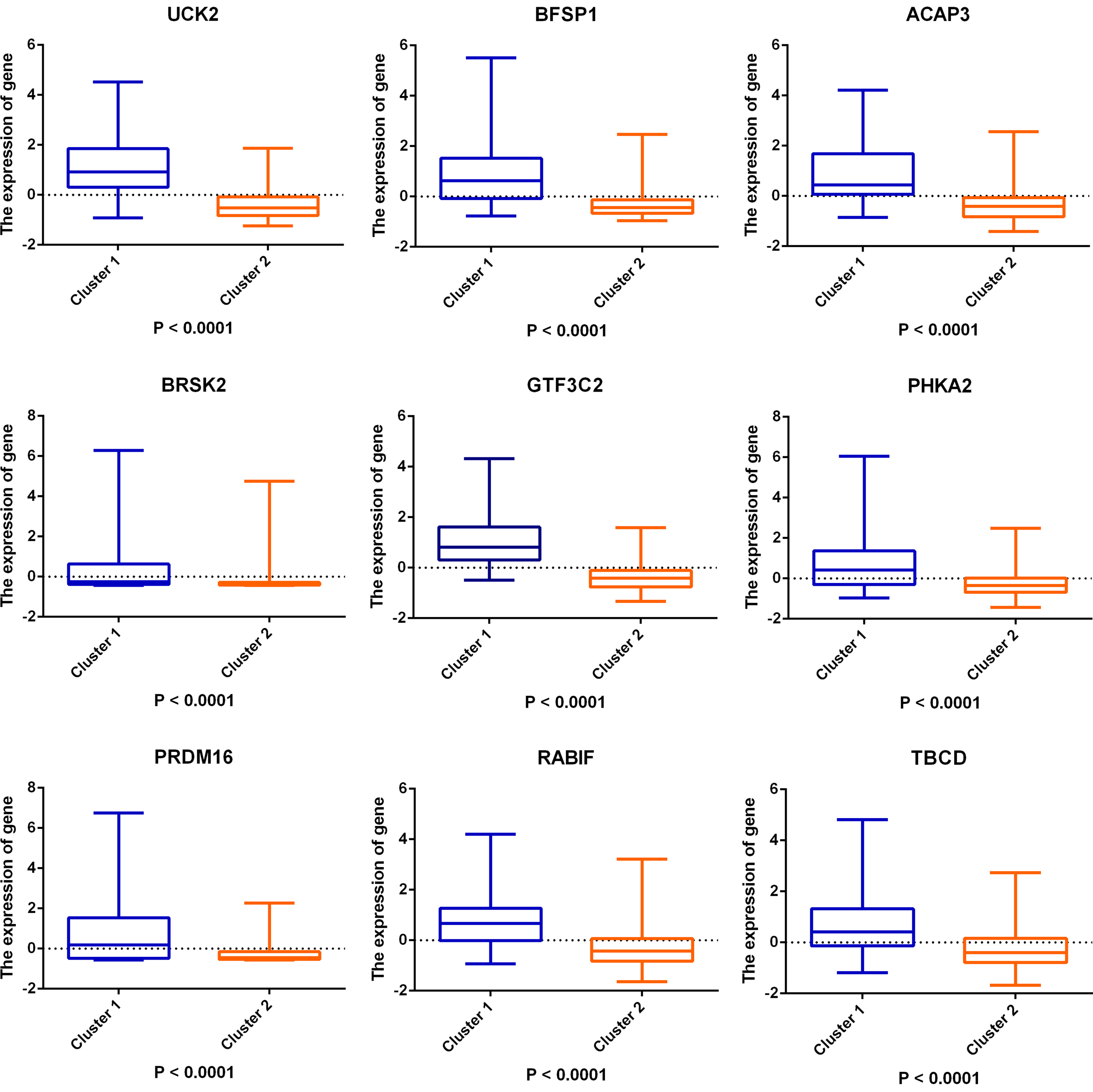

Supplement: Supplementary file 1 [file ijms-22-01474-s001.zip › Supplementary/Supplementary Figure S4.pdf]
